# Non-Isothermal Decomposition as Efficient and Simple Synthesis Method of NiO/C Nanoparticles for Asymmetric Supercapacitors

**DOI:** 10.3390/nano11010187

**Published:** 2021-01-13

**Authors:** Daria Chernysheva, Ludmila Pudova, Yuri Popov, Nina Smirnova, Olga Maslova, Mathieu Allix, Aydar Rakhmatullin, Nikolay Leontyev, Andrey Nikolaev, Igor Leontyev

**Affiliations:** 1 Platov South-Russian State Polytechnic University (NPI), 346428 Novocherkassk, Russia; da.leontyva@mail.ru (D.C.); pudowa.lyuda@yandex.ru (L.P.); smirnova_nv@mail.ru (N.S.); 2 Physics Department, Southern Federal University, 344090 Rostov-on-Don, Russia; popov@sfedu.ru; 3 Institute of Strength Physics and Materials Science, Siberian Branch, Russian Academy of Sciences, 346428 Tomsk, Russia; o_maslova@rambler.ru; 4 CNRS, CEMHTI UPR3079, Univ. Orléans, F-45071 Orléans, France; mathieu.allix@cnrs-orleans.fr (M.A.); aydar.rakhmatullin@cnrs-orleans.fr (A.R.); 5 Azov-Black Sea Engineering Institute, Don State Agrarian University, Rostov region, 347740 Zernograd, Russia; lng48@mail.ru; 6 Research and Education Center “Materials”, Don State Technical University, 344000 Rostov-on-Don, Russia; andreynicolaev@eurosites.ru

**Keywords:** supercapacitor, nickel oxide, NiO/C nanocomposite, non-isothermal decomposition

## Abstract

A series of NiO/C nanocomposites with NiO concentrations ranging from 10 to 90 wt% was synthesized using a simple and efficient two-step method based on non-isothermal decomposition of Nickel(II) bis(acetylacetonate). X-ray diffraction (XRD) measurements of these NiO/C nanocomposites demonstrate the presence of β-NiO. NiO/C nanocomposites are composed of spherical particles distributed over the carbon support surface. The average diameter of nickel oxide spheres increases with the NiO content and are estimated as 36, 50 and 205 nm for nanocomposites with 10, 50 and 80 wt% NiO concentrations, respectively. In turn, each NiO sphere contains several nickel oxide nanoparticles, whose average sizes are 7–8 nm. According to the tests performed using a three-electrode cell, specific capacitance (SC) of NiO/C nanocomposites increases from 200 to 400 F/g as the NiO content achieves a maximum of 60 wt% concentration, after which the SC decreases. The study of the NiO/C composite showing the highest SC in three- and two-electrode cells reveals that its SC remains almost unchanged while increasing the current density, and the sample demonstrates excellent cycling stability properties. Finally, NiO/C (60% NiO) composites are shown to be promising materials for charging quartz clocks with a power rating of 1.5 V (30 min).

## 1. Introduction

Asymmetric supercapacitors (SCs) are increasingly attracting attention as promising energy storage devices due to the constantly growing energy and ecological problems that are associated with fossil fuel depletion and global warming [1]. SCs contain transition metal oxides that are able to accumulate large charges through Faraday processes on the electrode surface. Among these oxides, NiO is of great interest [2,3] due to the high estimated capacitance (2584 F/g [4]), good corrosion resistance in alkaline solutions, and environmental safety.

Meanwhile, nickel oxide is known as a broadband p-type semiconductor (3.6–4.0 eV [5]) with a low electron conductivity (0.01–0.32 S/m [6]), which impedes its use in supercapacitor electrodes. This problem can be solved by reinforcing NiO’s conductivity with conducting carbon nanomaterials, such as ultradispersed soot, carbon nanotubes, or graphene, that possess highly developed surface, chemical inertness and high electron conductivity [7,8]. In comparison with pure nickel oxides, NiO/carbon composite structures ensure faster transfer of electron and ion charges, reduce the resistance of the rechargeable material and allow efficient application of the latter.

Electrodes of NiO supercapacitors are typically based on various nanostructures. On the one hand, the use of nanocrystalline NiO leads to an increase of the surface area. On the other hand, the size effect causes a decrease of the bandgap and an increase of conductivity [9,10]. The synthesis of nanometer scale NiO with different morphology, i.e., nano-sheets [11], thin film [12], plates [13], nanopyramids [14], and nanoparticles [15], is currently accessible via electrochemical routes [13,16], emulsion nanoreactor method [17], thermal decomposition of Ni(OH)_2_ at various temperatures [18,19], sol–gel [20], hydrothermal method [11,21], spray pyrolysis [12], etc.

Since the capacitance of oxide-based composites strongly depends on their microstructural characteristics that may differ at various synthesis conditions and component ratios, the development of an efficient and simple synthesis method that would ensure the production of composite electrode materials with optimal specific electrochemical properties is a relevant task. Furthermore, it was previously shown [22,23] that non-isothermal decomposition of Pt acetyl acetonate enables access to Pt/C nanoparticles whose surface area can reach 100 m^2^/g. This synthesis method is very simple and requires only two stages; moreover, no washing, filtering or subsequent drying of a specimen are required. Hence, the present study is based on the non-thermal decomposition synthesis for producing NiO/C nanoparticles. This synthesis was performed at constant heating rate, but at different nickel oxide-to-carbon carrier ratios in order to achieve the highest possible capacitance of a composite material. 

## 2. Materials and Methods

Nickel (II) acetylacetonate Ni(acac)_2_ (95%, Sigma-Aldrich, St. Louis, MO, USA) was used as a precursor for the synthesis of NiO/C nanocomposites, and Vulcan-XC 72 (Cabot, Boston, MA, USA) served as carbon support. At the first stage, nickel acetylacetonate was solved at room-temperature in Tetrahydrofuran solution (99.9%, Panreac Quimica SLU, Barcelona, Spain). The volume of solvent was chosen so that Ni(acac)_2_ was completely diluted. The carbon support was then added to the mixture using continuous stirring. After that, the blend was dried under continuous stirring using a magnetic stirrer and in an ultrasonic bath at a temperature of 50 °C. The powder was subsequently placed in a Nabertherm P300 high-temperature furnace (Nabertherm, Lilienthal, Germany). The synthesis was conducted under non-isothermal conditions in air at a heating rate of 1 K/min. The final synthesis temperature was determined from thermogravimetry data as 420 °C, which corresponded to complete acetylacetonate decomposition (Figure S1). According to the thermogravimetric data, nickel oxide concentrations in resulting composite materials were respectively 10, 20, 30, 40, 50, 60, 70, 80, and 90 wt%. 

The powder X-ray diffraction (XRD) data were collected on ARL X’TRA Thermo Fisher Scientific powder diffractometer (Thermo Electron SA, Ecublens, Switzerland). All XRD powder patterns were refined using the Rietveld method which enabled us to evaluate the average crystalline size D and parameter of the unit cell a. Since the β-form of nickel oxide has a space group Fm3m, the X-ray diffraction patterns were refined similarly to that described in the work [24,25]. The quality of X-ray refinement of NiO/C composite is shown in Figure S2. Scanning electron microscopy (SEM) images were obtained on a ZEISS Supra 25 unit microscope (Carl Zeiss SMT, Oberkochen, Germany) operated at 20 kV. Transmission electron microscopy (TEM) images obtained in a Philips CM 20 TEM microscope (Philips, Amsterdam, The Netherlands) operated at 200 kV. Raman spectra were measured at room temperature through 50× microscope objective using a Renishaw micro-Raman spectrometer (Renishaw plc., Gloucestershire, UK) equipped with argon laser (514.5 nm, max power Pex = 10 mW).

The electrochemical characterization of NiO/C composites as electrode materials for supercapacitors was performed at room temperature in a three-electrode cell using a P-150 potentiostat/galvanostat (Elins STC, Zelenograd, Moscow, Russia). A platinum wire and a saturated Ag/AgCl were used as counter and reference electrodes, respectively. The working electrodes were fabricated in accordance with the following procedure: 15 mg of a NiO/C composite (pure carbon black Vulcan XC-72) was suspended in a mixture of 2-propanol (Sigma-Aldrich) (0.5 mL) and 10 wt% aqueous Nafion^TM^ solution (Sigma-Aldrich) (0.02 mL). The mixture was stirred with a magnetic stirrer for 40 min; the formed ink was then dropped onto a glassy carbon foil electrode (Sigma-Aldrich) and was air-dried. The amount of dried ink in the working electrode was 1.0 mg. A 1 M KOH aqueous solution was used as electrolyte. Cyclic voltammetry measurements (CVs) were conducted in a potential range of −0.1 to 0.65 V at different scan rates. The galvanostatic charge–discharge (GCD) and cycle-life/stability studies were carried out within the potential range of 0 to 0.5 V. Based on the GCD process, the specific capacitance (*C*_s_) was obtained from the following equation [26]:

$$C_{s}=\;\frac{I\;\times \;\Delta t}{m\;\times \;\Delta V}$$

where *C*_s_ (F g^−1^) is the specific capacitance in a three-electrode system; *I* (A) is the discharging current; Δ*t* (s) is the discharge time; *m* (g) is the weight of active materials (NiO/C composite or pure carbon black Vulcan XC-72); and Δ*V* (V) is the voltage charge during the discharge process. 

A home-made cell of asymmetrical two-electrode SCs was assembled from polytetrafluoroethylene, where positive and negative current collectors were made of a nickel foil (23 mm in diameter). The separator consisted of a hydrophilic polypropylene membrane, and the electrolyte was a 6 M KOH solution. Both electrodes were prepared by mixing the active electrode material (NiO/C nanocomposite in the case of the positive electrode and carbon black (CB) Timcal Super C65 (Nanografi Nano Technology, Ankara, Turkey) for the negative electrode), the CB conductor (Timcal Super C65) and polyvinylidene difluoride (PVDF) with weight ratio of 7.5:1:1.5. A few drops of N-Methylpyrrolidone (Sigma-Aldrich) were added to form the slurry that was smeared onto the nickel foil current collector and exposed to 6 h of drying at 80 °C.

The electrochemical performance of the two-electrode system was tested via CV and GCD measurements. CV measurements were carried out in a potential range of 0 to 1.65 V at different scan rates. The GCD and cycle-life/stability studies were implemented in the same potential range. In the context the GCD process, the specific capacitance of a two-electrode system was obtained, as follows [27]:

$$C_{s}=\;\frac{I\;\times \;\Delta t}{M\;\times \;\Delta V}$$

where *C*_s_ (F g^−1^) is the specific capacitance of a two-electrode system; *I*(A) is the discharging current; Δ*t* (s) is the discharge time; *M*(g) is the total weight of active materials on positive and negative electrodes; and Δ*V* (V) is the voltage charge during the discharge process.

The mass ratio between the positive and negative electrodes can be found using the charge balance (q^+^ = q^−^) from the equation below [26,27]:

$$\frac{m_{+}}{m_{-}}=\;\frac{C_{-}\;\times \;\Delta V_{-}}{C_{+}\;\times \;\Delta V_{+}}$$

where *m* is the mass, *C* is the specific capacitance, and Δ*V* is the potential window of the electrode. The electrochemical properties of the NiO/C composite and CB in a three-electrode system were examined separately through the CV and GCD measurements. According to the peculiar electrochemical behavior of each component, the optimal loading mass ratio of NiO/C to CB is about 0.045.

## 3. Results and Discussion

**Figure 1:**
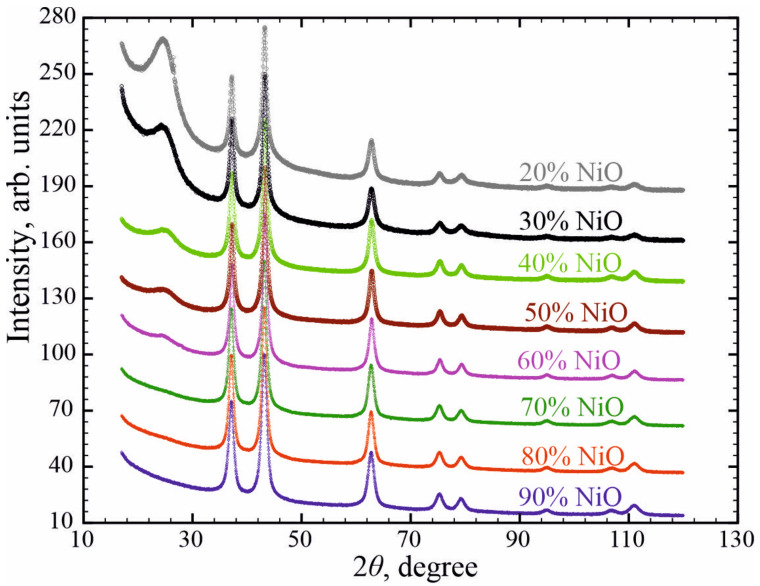
X-ray powder diffraction patterns of freshly prepared NiO/C nanocomposites.

**Figure 2:**
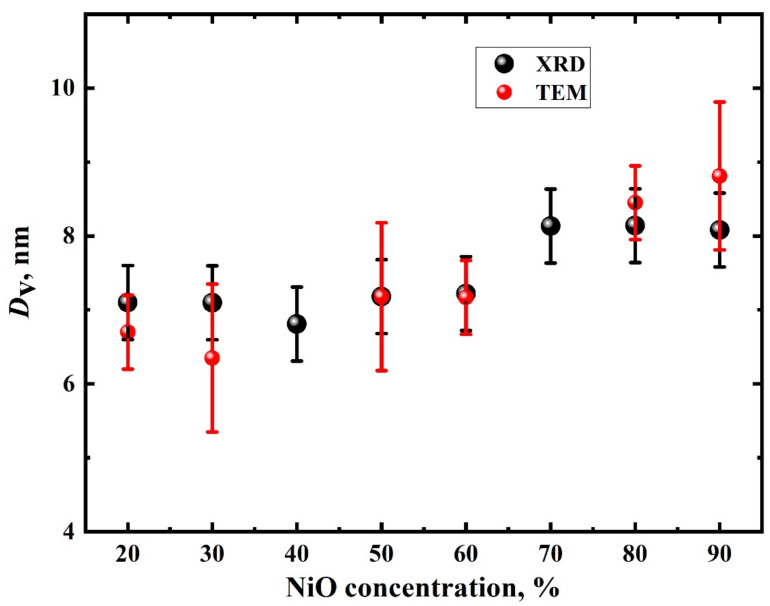
Nanoparticle average sizes evaluated from XRD (black circles) and TEM (red circles) data.

Figure 1 displays the data for all NiO/C composites. The peaks correspond to β-NiO (4.18 Å, *Fm*3*m*). Besides this, the 2*θ* angle range of NiO-deficient composites exhibits the presence of a broadened peak referred to a Vulcan carbon support. The average NiO nanoparticle size as a function of concentration, determined by XRD data refinement of NiO/C composites, evidences a slight increase in nanoparticle size from 7 to 8 nm while increasing the NiO concentration (Figure 2). The unit cell parameters vary from 4.1830 Å to 4.1792 Å when increasing the NiO concentration from 10 to 90 wt%, which exceeds the unit cell parameter *a* = 4.1771 Å for bulk NiO (JCPDS card no. 78-0429). A similar effect of the unit cell parameter behaving as an inverse relationship of the average NiO nanoparticle size was also observed in previous works [10].

Each spectrum can be roughly divided into three areas: (i) 300…1200 cm^−1^, region where standard modes of NiO are observed (TO, LO, 2TO, TO+LO, 2LO) (Figure S3); (i) 1200…1800 cm^−1^, the region where the Vulcan modes are observed, corresponding to the first scattering order and representing a pattern typical for amorphous carbon (bands D and G); in this case, the two-magnon mode of NiO (~1490 cm^−1^) is not visible (Figures S4 and S5).

Spectra can be conventionally divided into two groups: (i) spectra of samples with NiO concentration of 10%, 20%, 30%, 50% and 80%—in these spectra the Vulcan peaks are most pronounced and the NiO peaks are weakly visible (Figure S4); (ii) spectra of samples with NiO concentration of 40%, 60%, 70%, 90%—in these spectra the NiO peaks are most pronounced and the Vulcan peaks are weakly visible (Figure S5). 

**Figure 3:**
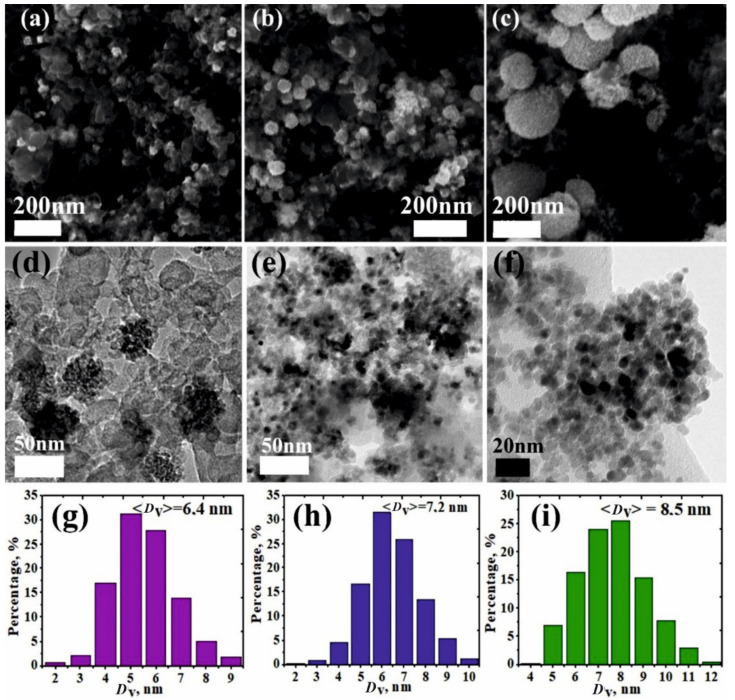
SEM (**a**–**c**) and TEM (**d**–**f**) images and grain size distributions (**g**–**i**) of NiO nanoparticles in NiO/C nanocomposites with different NiO concentrations: (**a**,**d**,**g**) 10 wt%, (**b**,**e**,**h**) 50 wt%, (**c**,**f**,**i**) 90 wt%.

Scanning and transmission electron microscopy data of the synthesized NiO/C samples are plotted in Figure 3. As seen from the SEM images (Figure 3a,c), the samples are composed of NiO spherical particles distributed over the carbon support surface. Their sizes increase with NiO concentration in the produced nanocomposites. The average diameters of nickel oxide spheres are found to be 36 nm, 50 nm and 205 nm for nanocomposites with 10, 50 and 80 wt% of NiO concentrations, respectively. Based on these XRPD results, each sphere contains individual nickel oxide nanoparticles. This conclusion is consistent with TEM optical micrographs of NiO/C composites (Figure 3d–f). 

Figure 3g–i illustrate the particle size distributions at various NiO concentrations in composites. The average NiO nanoparticle size as a function of NiO concentration is given in Figure 2. It is evident that the average nanoparticle sizes evaluated from TEM data is commensurate with those estimated via the Rietweld method.

**Figure 4:**
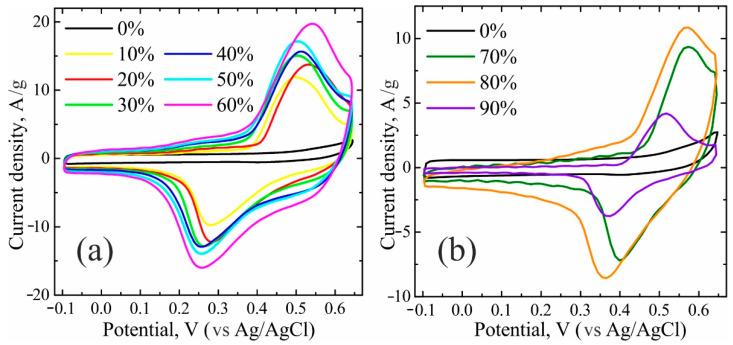
Cyclic voltammograms (CVs) of NiO/C samples, divided into two groups with NiO concentrations of: (**a**): 0, 10, 20, 30, 40, 50, 60 wt%; (**b**) 0, 70, 80, 90 wt%.

**Figure 5:**
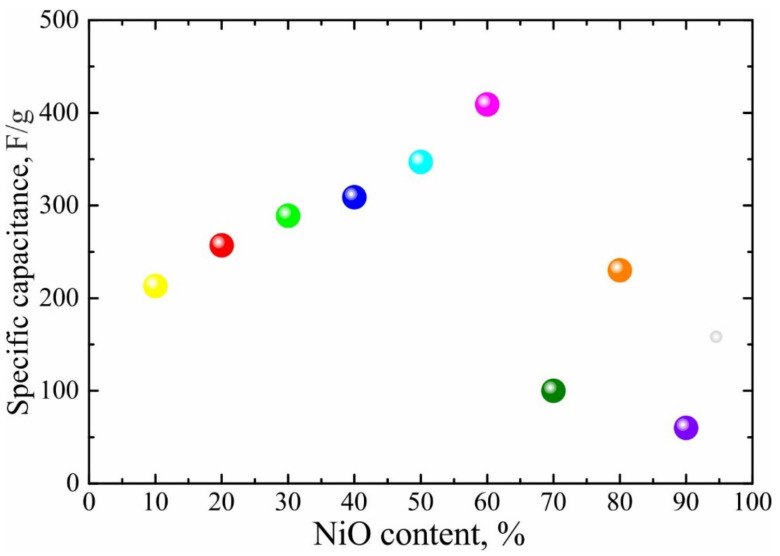
Specific capacitance as a function of NiO content in NiO/C composites at a current density of 0.5 F g^−1^ in a three-electrode cell in a 1 M KOH solution. The results are divided into two groups.

The electrochemical results of all NiO/C composites, obtained in a three-electrode cell, are plotted in Figure 4 and Figure 5. The cyclic voltammograms (CVs) can be divided into two groups with respect to their profiles (Figure 4). The first group comprises the composites with nickel oxide contents ranging from 10 to 60 wt% inclusively (Figure 4a). The current peaks of the higher nickel oxide formation at potentials unfolding towards the anodic range, as well as the appropriate current peaks of the higher nickel oxide reduction upon the potential unfolding towards the cathodic range in a series from 10 wt% to 60 wt%, increase regularly with the NiO amount, and all CVs exhibit an analogous conventional shape. The second group (Figure 4b), including NiO/C composites with NiO concentrations from 70 to 90 wt%, evidences no regularities from the first group. Along with galvanostatic data for all composites, displayed in Figure 5, such a division into two groups can be explained in the context of the percolation theory in strongly inhomogeneous media [28]. NiO/C composites are composed of a low-conductivity semiconducting component of nickel oxide (10^−9^ S cm^−1^ [9]) and an electron conductor of Vulcan carbon soot at their certain ratio, which ensures sufficient conductivity. According to earlier studies [29], starting from the volume soot concentration of 65 vol% (which corresponds to ~40 wt%), the electron conductivity of a composite becomes commensurate to that of pure soot, which allows the electrochemical data to be interpreted in a correct manner.

Preliminary examination in a three-electrode cell enables to conclude that a NiO/C composite with a 60 wt% NiO concentration possesses the best electrochemical properties, which makes it a desirable object for more comprehensive investigation of electrochemical characteristics.

**Figure 6:**
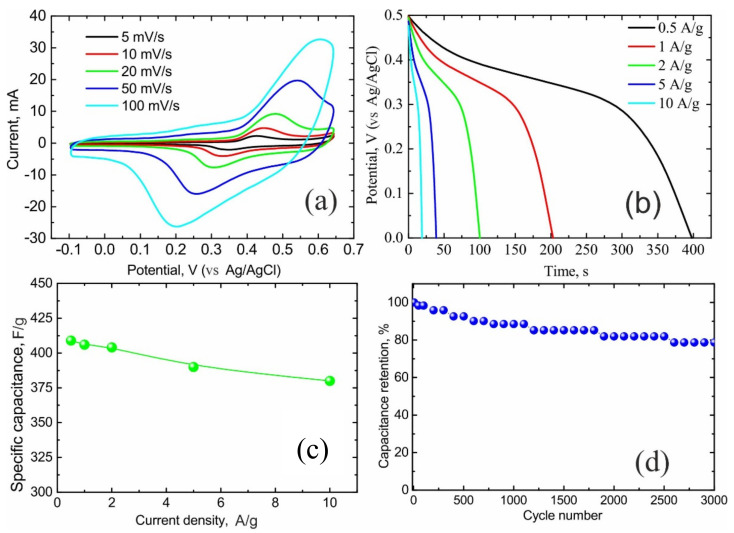
Electrochemical performance of NiO/C nanocomposite (60 wt% NiO) in a three-electrode cell in a 1 M KOH aqueous solution: (**a**) CVs at various scan rates; (**b**) galvanostatic discharge curves at various current densities; (**c**) specific capacitance as a function of current density; (**d**) cycling stability at the current density of 2 A/g.

The CVs of NiO/C (60 wt%) samples (Figure 6a) exhibit a pronounced anodic peak, associated with a higher oxide formation on the surface, and a cathodic peak of their reduction described by the following Equation (1):

NiO + OH¯ ⟷ NiOOH + e¯(1)

The maximum current of the anodic peak depends on the potential unfolding rate, meaning that the diffusion limiting process is the proton diffusion in the solid phase.

In order to gather more detailed information on the specific capacitance of NiO/C (60 wt%) composite, the galvanostatic charge/discharge measurements were made at various current densities (Figure 6b). As clearly seen in Figure 6c, the specific capacitance remains almost unchanged while increasing the current density.

In the Table S1 demonstrated a comparation of the electrochemical performance of the obtained NiO/C (60 wt% NiO) and other NiO/C composites with different carbon supports (composite microspheres [30], hollow microspheres [31], CNTs [32], carbon coating on the NiO [33], graphite [34], sulfonated graphene [35], carbon nanospheres [36]), prepared by various methods [30,31,32,33,34,35,36]. One can see that the *C*_s_ of the NiO/C (60 wt% NiO) at the current density of 1 A g^−1^ is 405 Fg^−1^ (Figure 6c, Table S1) which is nearly the same [35,36] or much higher than *C*_s_ [30,31,32,33,34] of other materials.

**Figure 7:**
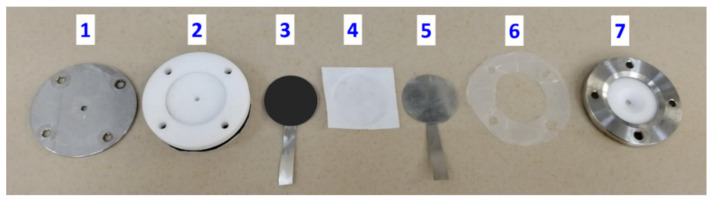
A two-electrode cell: 1—stainless steel plate, 2—polytetrafluoroethylene cell, 3, 5—current collectors (Ni foil), 4—separator (hydrophilic polypropylene membrane), 6—polytetrafluoroethylene isolation gasket, 7—stainless steel cover with polytetrafluoroethylene spacer.

In order to investigate the electrochemical properties of NiO/C nanocomposite (60 wt% NiO) in terms of its potential applications, an asymmetric two-electrode cell was assembled (Figure 7) with a positive electrode of NiO/C (60 wt% NiO) and a negative electrode of carbon black Timcal Super C65 in a 6 M KOH electrolyte.

**Figure 8:**
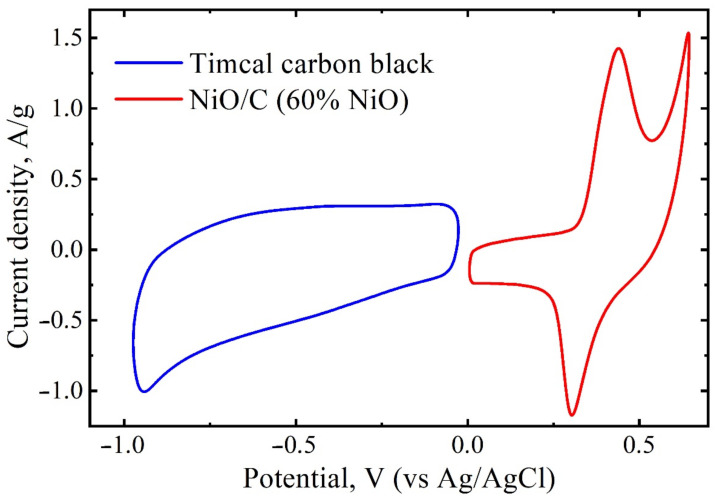
CV curves of carbon black Timcal Super C65 (CB) and NiO/C (60 wt% NiO) in a three-electrode cell in a 6 M KOH solution at a scan rate of 50 mV s^−1^.

The use of such electrodes conforms to the principle of constructing an asymmetric supercapacitor, because the potential windows for carbon and NiO/C electrodes are −1–0 V and 0–0.65 V versus a saturated Ag/AgCl electrode, respectively (Figure 8), which helps widen the work potentials of the cell. 

**Figure 9:**
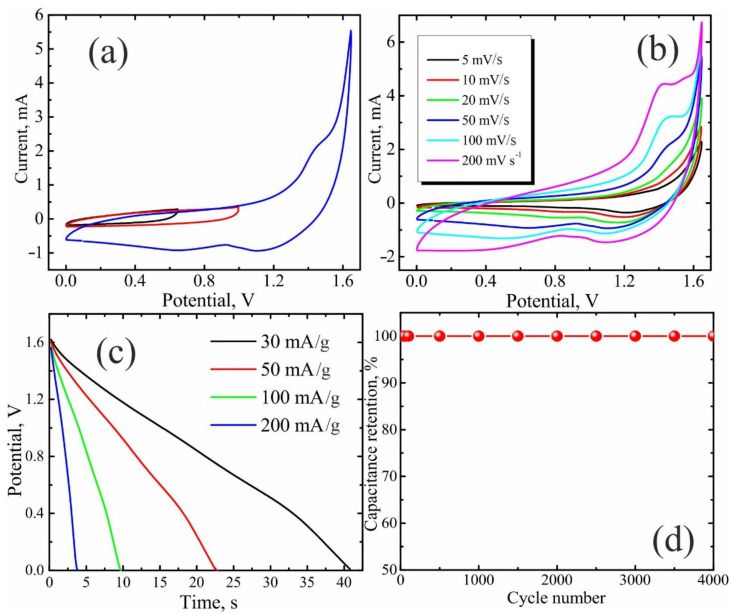
Electrochemical performance of the asymmetrical two-electrode cell with carbon black Timcal Super C65 and NiO/C (60 wt% NiO) as negative and positive electrodes, respectively, in a 6 M KOH solution: (**a**) cyclic voltammogram (CV) curves within different potential ranges up to 0.65, 1 and 1.65 V at 50 mV s^−1^; (**b**) CV curves at various scan rates; (**c**) galvanostatic discharge curves at various current densities; (**d**) cycling stability at a current density of 0.1 A g^−1^ during 4000 cycles.

A series of CV curves were measured within different potential ranges at a scan rate of 50 mV/s to estimate the optimal potential window for the asymmetrical two-electrode cell (Figure 9a). When the potential window increased from 1 to 1.65 V, two redox peaks were observed, indicating the pseudocapacitive behavior of the supercapacitor. Based on these results, the CV measurements were performed at different scan rates within a potential window of 1.65 V. As shown in Figure 9b the CV curves of the assembled cell maintained their shape even at high scan rates, evidencing its good rate capability. 

The galvanostatic discharge curves exhibit no IR drop due to the internal resistance in the initial range of a discharge curve (Figure 9c). The several-day-long cycling stability tests of the asymmetric two-electrode cell (4000 cycles under a current density of 0.1 A g^−1^) reveal the excellent cyclic performance and the enduring stability (Figure 9d), which is essential for practical applications.

**Figure 10:**
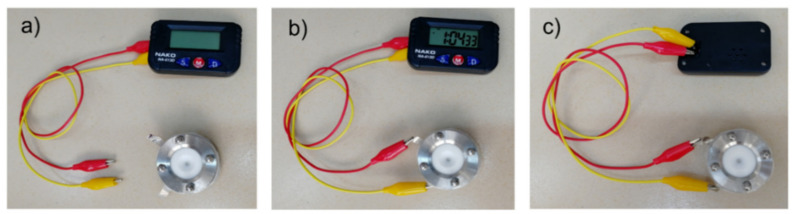
Application of the asymmetrical two-electrode assembled cell (NiO/C (60 wt% NiO) as a positive electrode and CB Timcal Super C65 as a negative electrode) in a 6 M KOH aqueous solution: (**a**) photograph of an asymmetrical two-electrode assembled cell charged at 0.5 mA and a battery-free quartz clock; (**b**) photograph of a quartz clock (front side) in the process of charging; (**c**) photograph of a quartz clock (back side) in the process of charging.

The voltage supplied by a single asymmetrical two-electrode cell with a NiO/C positive electrode (60 wt% NiO) and an aqueous electrolyte is sufficient for the use in different micro-devices, for example, in powering quartz clocks with a power rating of 1.5 V for about 30 min (Figure 10).

## Data Availability

The data presented in this study are available on request from the corresponding author.

## Supplementary Materials

The following are available online at https://www.mdpi.com/2079-4991/11/1/187/s1, Figure S1: TGA curve of Ni(acac)_2_: thermal decomposition at a heating rate of 1 K/min under air atmosphere., Figure S2: Experimental (red dots) and simulated (black solid line) XRD patterns of NiO/C nanocomposite (80 wt% NiO). The blue line is the difference between experimental and theoretical XRD patterns. The tick marks correspond to the Bragg peak positions of fcc *Fm3m* phase of NiO (Agreement factors were R_p_ = 4.77%, R_wp_ = 3.82%, and R_exp_ = 2.67%). Figure S3: Decomposition into components of the NiO/Vulcan spectrum (NiO concentration—80%). Empty circles and blue curve correspond to experimental data, green curves—to NiO peaks, gray curves—to Vulcan peaks. Figure S4: The first group of NiO/Vulcan spectra. NiO concentrations: 10%, 20%, 30%, 50%, 80%. Figure S5: The second group of NiO/Vulcan spectra. NiO concentrations: 40%, 60%, 90%, 95%. Table S1: Comparison of electrochemical performances of NiO/C (60 wt% NiO) and various NiO/C composites) in a three-electrode system (alkaline aqueous solution).

## References

[1] Lu X., Yu M., Wang G., Tong Y., Li Y. (2014). Flexible Solid-State Supercapacitors: Design, Fabrication and Applications. Energy Environ. Sci..

[2] Sk M.M., Yue C.Y., Ghosh K., Jena R.K. (2016). Review on Advances in Porous Nanostructured Nickel Oxides and Their Composite Electrodes for High-Performance Supercapacitors. J. Power Sources.

[3] Paulose R., Mohan R., Parihar V. (2017). Nanostructured Nickel Oxide and Its Electrochemical Behaviour—A Brief Review. Nano Struct. Nano Objects.

[4] Liu K.-C., Anderson M.A. (1996). Porous Nickel Oxide/Nickel Films for Electrochemical Capacitors. J. Electrochem. Soc..

[5] Sasi B., Gopchandran K., Manoj P., Koshy P., Prabhakara Rao P., Vaidyan V. (2002). Preparation of Transparent and Semiconducting NiO Films. Vacuum.

[6] Nandy S., Maiti U.N., Ghosh C.K., Chattopadhyay K.K. (2009). Enhanced P-Type Conductivity and Band Gap Narrowing in Heavily Al Doped NiO Thin Films Deposited by RF Magnetron Sputtering. J. Phys. Condens. Matter.

[7] Li Q., Li C.-L., Li Y.-L., Zhou J.-J., Chen C., Liu R., Han L. (2017). Fabrication of Hollow N-Doped Carbon Supported Ultrathin NiO Nanosheets for High-Performance Supercapacitor. Inorg. Chem. Commun..

[8] Lokhande V.C., Lokhande A.C., Lokhande C.D., Kim J.H., Ji T. (2016). Supercapacitive Composite Metal Oxide Electrodes Formed with Carbon, Metal Oxides and Conducting Polymers. J. Alloys Compd..

[9] Makhlouf S.A., Kassem M.A., Abdel-Rahim M.A. (2009). Particle Size-Dependent Electrical Properties of Nanocrystalline NiO. J. Mater. Sci..

[10] Yazdani A., Zafarkish H., Rahimi K. (2018). The Variation of Eg-Shape Dependence of NiO Nanoparticles by the Variation of Annealing Temperature. Mater. Sci. Semicond. Process..

[11] Bose P., Ghosh S., Basak S., Naskar M.K. (2016). A Facile Synthesis of Mesoporous NiO Nanosheets and Their Application in CO Oxidation. J. Asian Ceram. Soc..

[12] Ukoba K.O., Eloka-Eboka A.C., Inambao F.L. (2018). Review of Nanostructured NiO Thin Film Deposition Using the Spray Pyrolysis Technique. Renew. Sustain. Energy Rev..

[13] Aghazadeh M. (2017). Synthesis, Characterization, and Study of the Supercapacitive Performance of NiO Nanoplates Prepared by the Cathodic Electrochemical Deposition-Heat Treatment (CED-HT) Method. J. Mater. Sci. Mater. Electron..

[14] Kumar A., Sanger A., Kumar A., Chandra R. (2017). Single-Step Growth of Pyramidally Textured NiO Nanostructures with Improved Supercapacitive Properties. Int. J. Hydrogen. Energy.

[15] Lontio Fomekong R., Ngolui Lambi J., Ebede G.R., Kenfack Tsobnang P., Tedjieukeng Kamta H.M., Ngnintedem Yonti C., Delcorte A. (2016). Effective Reduction in the Nanoparticle Sizes of NiO Obtained via the Pyrolysis of Nickel Malonate Precursor Modified Using Oleylamine Surfactant. J. Solid State Chem..

[16] Leontyeva D.V., Leontyev I.N., Avramenko M.V., Yuzyuk Y.I., Kukushkina Y.A., Smirnova N.V. (2013). Electrochemical Dispergation as a Simple and Effective Technique toward Preparation of NiO Based Nanocomposite for Supercapacitor Application. Electrochim. Acta.

[17] Fazlali F., Mahjoub A., Abazari R. (2015). A New Route for Synthesis of Spherical NiO Nanoparticles via Emulsion Nano-Reactors with Enhanced Photocatalytic Activity. Solid State Sci..

[18] El-Kemary M., Nagy N., El-Mehasseb I. (2013). Nickel Oxide Nanoparticles: Synthesis and Spectral Studies of Interactions with Glucose. Mater. Sci. Semicond. Process..

[19] Motahari F., Mozdianfard M.R., Soofivand F., Salavati-Niasari M. (2014). NiO Nanostructures: Synthesis, Characterization and Photocatalyst Application in Dye Wastewater Treatment. RSC Adv..

[20] Danial A.S., Saleh M.M., Salih S.A., Awad M.I. (2015). On the Synthesis of Nickel Oxide Nanoparticles by Sol–Gel Technique and Its Electrocatalytic Oxidation of Glucose. J. Power Sources.

[21] Purushothaman K.K., Manohara Babu I., Sethuraman B., Muralidharan G. (2013). Nanosheet-Assembled NiO Microstructures for High-Performance Supercapacitors. ACS Appl. Mater. Interfaces.

[22] Kulbakov A.A., Allix M., Rakhmatullin A., Mikheykin A.S., Popov Y.V., Smirnova N.V., Maslova O., Leontyev I.N. (2018). In Situ Investigation of Non-Isothermal Decomposition of Pt Acetylacetonate as One-Step Size-Controlled Synthesis of Pt Nanoparticles. Phys. Status Solidi.

[23] Kulbakov A.A., Kuriganova A.B., Allix M., Rakhmatullin A., Smirnova N., Maslova O.A., Leontyev I.N. (2018). Non-Isothermal Decomposition of Platinum Acetylacetonate as a Cost-Efficient and Size-Controlled Synthesis of Pt/C Nanoparticles. Catal. Commun..

[24] Leontyev I.N., Kuriganova A.B., Allix M., Rakhmatullin A., Timoshenko P.E., Maslova O.A., Mikheykin A.S., Smirnova N.V. (2018). On the Evaluation of the Average Crystalline Size and Surface Area of Platinum Catalyst Nanoparticles. Phys. Status Solidi Basic Res..

[25] Leontyev I.N., Kulbakov A.A., Allix M., Rakhmatullin A., Kuriganova A.B., Maslova O.A., Smirnova N.V. (2017). Thermal Expansion Coefficient of Carbon-Supported Pt Nanoparticles: In-Situ X-Ray Diffraction Study. Phys. Status Solidi Basic Res..

[26] Chernysheva D., Vlaic C., Leontyev I., Pudova L., Ivanov S., Avramenko M., Allix M., Rakhmatullin A., Maslova O., Bund A. (2018). Synthesis of Co_3_O_4_/CoOOH via electrochemical dispersion using a pulse alternating current method for lithium-ion batteries and supercapacitors. Solid State Sci..

[27] Liu S., Lee S.C., Patil U.M., Ray C., Sankar K.V., Zhang K., Kundu A., Kang S., Park J.H., Jun S.C. (2017). Controllable Sulfuration Engineered NiO Nanosheets with Enhanced Capacitance for High Rate Supercapacitors. J. Mater. Chem. A.

[28] Shklovskii B.I., Éfros A.L. (1975). Percolation Theory and Conductivity of Strongly Inhomogeneous Media. Uspekhi Fiz. Nauk.

[29] Kuriganova A.B., Vlaic C.A., Ivanov S., Leontyeva D.V., Bund A., Smirnova N.V. (2016). Electrochemical Dispersion Method for the Synthesis of SnO_2_ as Anode Material for Lithium Ion Batteries. J. Appl. Electrochem..

[30] Wang Y., Xing S., Zhang E., Wei J., Suo H., Zhao C., Zhao X. (2012). One-pot synthesis of nickel oxide–carbon composite microspheres on nickel foam for supercapacitors. J. Mater. Sci..

[31] Liu J., Wickramaratne N.P., Qiao S.Z., Jaroniec M. (2015). Molecular-based design and emerging applications of nanoporous carbon spheres. Nat. Mater..

[32] Sannasi V., Maheswari K.U., Karthikeyan C., Karuppuchamy S. (2020). H_2_O_2_-assisted microwave synthesis of NiO/CNT nanocomposite material for supercapacitor applications. Ionics.

[33] Vijayakumar S., Nagamuthu S., Muralidharan G. (2013). Porous NiO/C Nanocomposites as Electrode Material for Electrochemical Supercapacitors. ACS Sustain. Chem. Eng..

[34] Wu S.R., Liu J.B., Wang H., Yan H. (2019). NiO@graphite carbon nanocomposites derived from Ni-MOFs as supercapacitor electrodes. Ionics.

[35] Wang L., Tian H., Wang D., Qin X., Shao G. (2015). Preparation and electrochemical characteristic of porous NiO supported by sulfonated graphene for supercapacitors. Electrochim. Acta.

[36] Liu M., Wang X., Zhu D., Li L., Duan H., Xu Z., Wang Z., Gan L. (2017). Encapsulation of NiO nanoparticles in mesoporous carbon nanospheres for advanced energy storage. Chem. Eng. J..

