# Supplementary material for: Non-Isothermal Decomposition as Efficient and Simple Synthesis Method of NiO/C Nanoparticles for Asymmetric Supercapacitors
Source: Nanomaterials (Basel). 2021 Jan 13;11(1):187. doi: 10.3390/nano11010187 (PMC7828437; doi:10.3390/nano11010187)
Supplement: Supplementary file 1 [file nanomaterials-11-00187-s001.pdf]

nanomaterials

*Supplementary Materials*

# Non-Isothermal Decomposition as Efficient and Simple Synthesis Method of NiO/C Nanoparticles for Asymmetric Supercapacitors

**Daria Chernysheva [1], Ludmila Pudova [1], Yuri Popov [2], Nina Smirnova [1], Olga Maslova [3], Mathieu Allix [4], Aydar Rakhmatullin [4], Nikolay Leontyev [5], Andrey Nikolaev [6] and Igor Leontyev [2,*]**

1 Platov South-Russian State Polytechnic University (NPI), 346428, Novocherkassk, Russia; da.leontyva@mail.ru (D.C.); pudowa.lyuda@yandex.ru (L.P.); smirnova_nv@mail.ru (N.S.)
2 Southern Federal University, 344090 Rostov-on-Don, Russia; popov@sfedu.ru
3 Institute of Strength Physics and Materials Science, Siberian Branch, Russian Academy of Sciences, 346428 Tomsk, Russia; o_maslova@rambler.ru
4 CNRS, CEMHTI UPR3079 Univ. CEDEX F-45071 Orléans, France; mathieu.allix@cnrs-orleans.fr (M.A.); aydar.rakhmatullin@cnrs-orleans.fr (A.R.)
5 Azov-Black Sea Engineering Institute, Don State Agrarian University, Rostov region, 347740 Zernograd, Russia; lng48@mail.ru
6 Research and Education Center "Materials", Don State Technical University, 344000 Rostov-on-Don, Russia; andreynicolaev@eurosites.ru
* Correspondence: i.leontiev@rambler.ru; Tel.: +7-918-552-4024

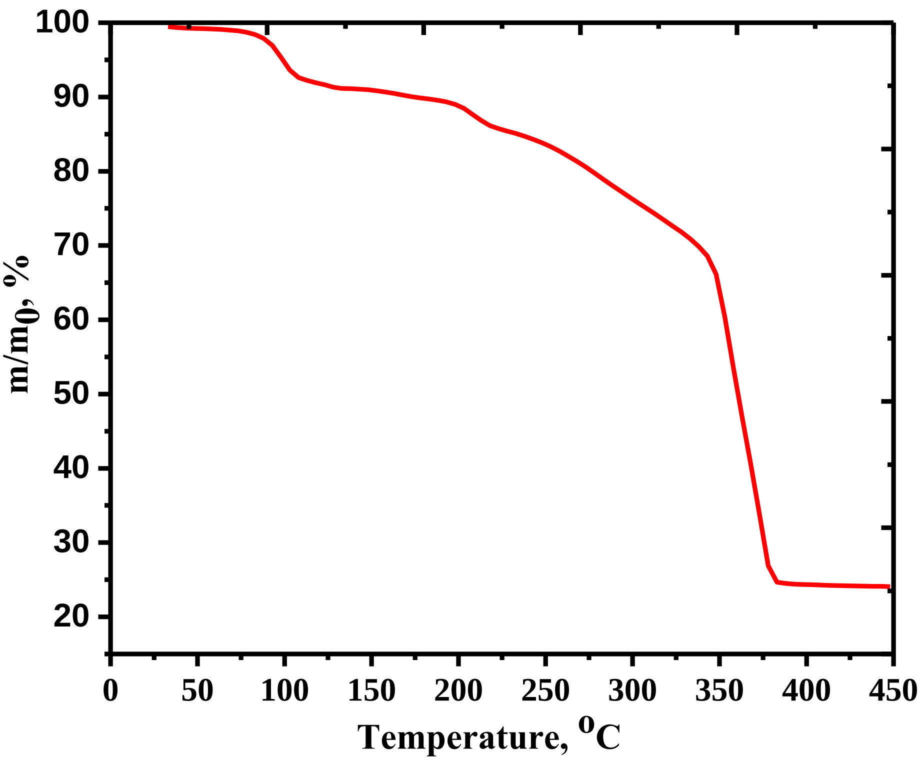


**Figure S1.** TGA curve of Ni(acac)$_2$: thermal decomposition at a heating rate of 1 K/min under air atmosphere.

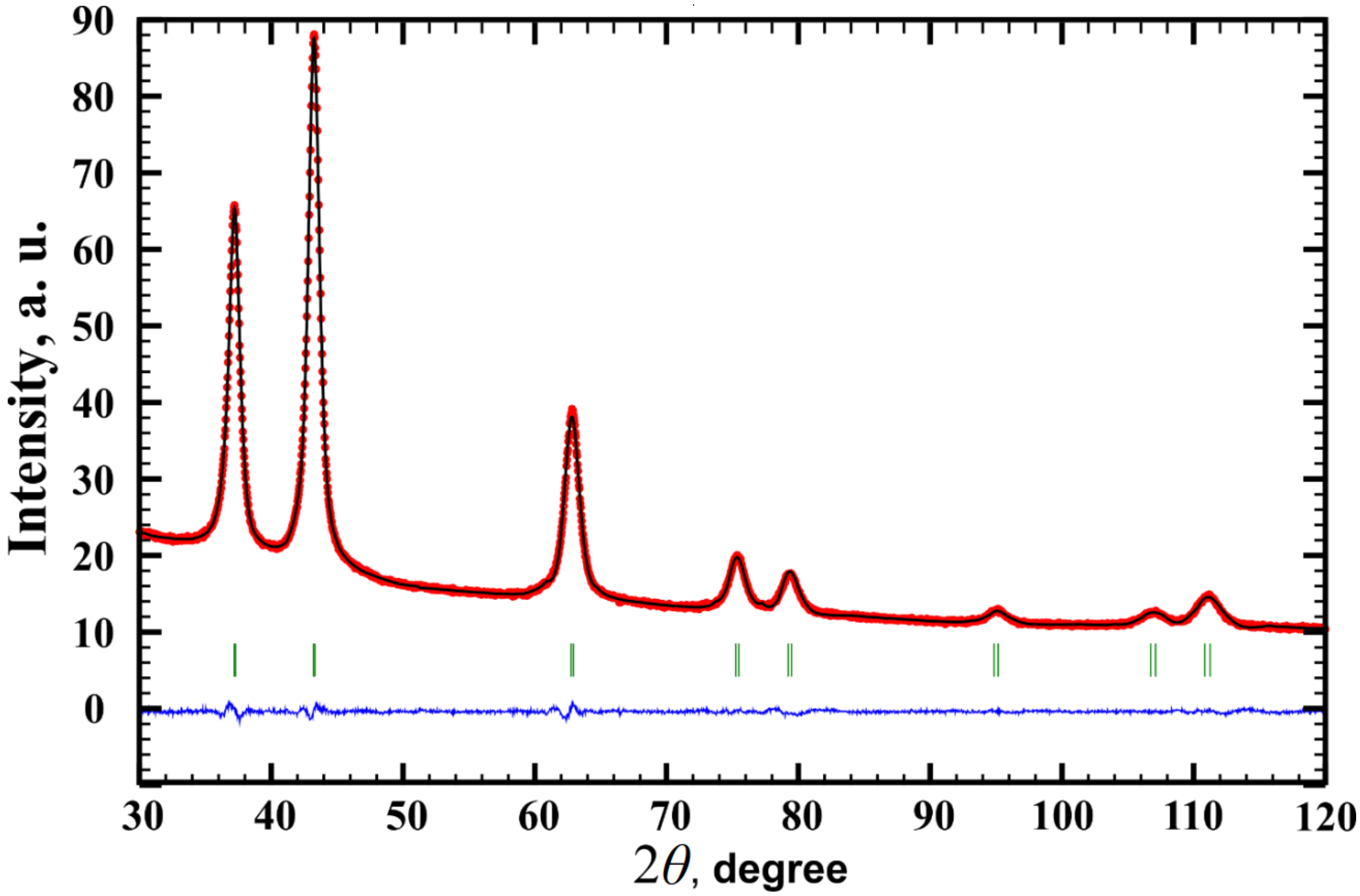


**Figure S2.** Experimental (red dots) and simulated (black solid line) XRD patterns of NiO/C nanocomposite (80 wt% NiO). The blue line is the difference between experimental and theoretical XRD patterns. The tickmarks correspond to the Bragg peak positions of fcc *Fm3m* phase of NiO (Agreement factors were $R_p$ = 4.77%, $R_{wp}$ = 3.82%, and $R_{exp}$ = 2.67%).

**Table S1.** Comparison of electrochemical performances of NiO/C (60 wt% NiO) and various NiO/C composites in a three-electrode system (alkaline aqueous solution).

| Sample | Cs, F $g^{-1}$ at 1 A $g^{-1}$ | Cycling Performance | Ref. |
|---|---|---|---|
| NiO/C (60% wt% NiO) | 405 F $g^{-1}$ | 80% retention after 3000 cycles at 2 A $g^{-1}$ | this work |
| NiO-C-nickel foam | 83.2 F $g^{-1}$ | 93.04% retention after 1000 cycles at 0.25 A $g^{-1}$ | [30] |
| Double-shelled C@NiO hollow microspheres | 211 F $g^{-1}$ | 96% retention after 1000 cycles at 5 A $g^{-1}$ | [31] |
| NiO/CNTs | 258 F $g^{-1}$ | 86% retention after 2500 cycles at 6 A $g^{-1}$ | [32] |
| NiO/C-5 | 250 F $g^{-1}$ | 88% retention after 1000 cycles at 10 mA $cm^{-2}$ | [33] |
| NiO/graphite (pyrolysis of Ni-MOFs at 300 °C) | 317 F $g^{-1}$ | 74% retention after 1500 cycles at 2 A $g^{-1}$ | [34] |
| NiO/sulfonated graphene | 385 F $g^{-1}$ | 90% retention after 1000 cycles at 5.0 A $g^{-1}$ | [35] |
| NiO nanoparticles in carbon nanospheres | 406 F $g^{-1}$ | 91% retention after 10,000 cycles at 3 A $g^{-1}$ | [36] |

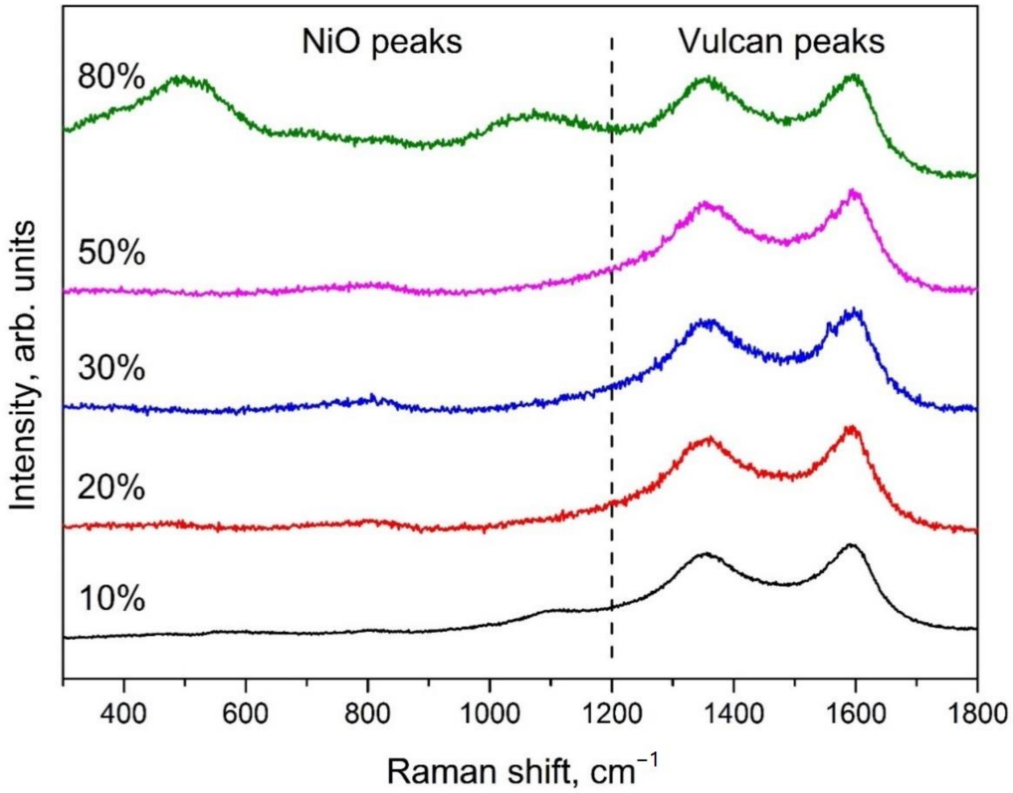


**Figure S3.** The first group of NiO / Vulcan spectra. NiO concentrations: 10, 20, 30, 50, 80%.

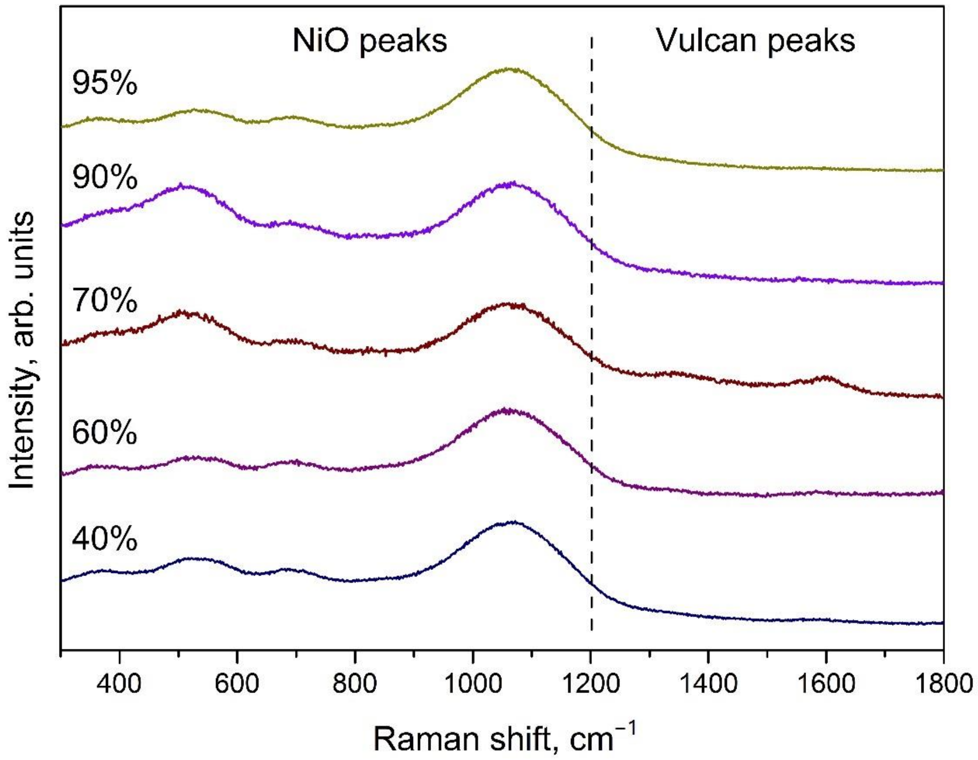


**Figure S4.** The second group of NiO / Vulcan spectra. NiO concentrations: 40, 60, 90, 95%.

Each spectrum was decomposed into its components using the Lorentz function. An example of this decomposition is shown in Figure S5. As a result of the spectra decomposition analysis, it was revealed that the frequencies and full width at half maximum (FWHM)of the peaks do not have any pronounced dependence on the NiO concentration in the samples. On the other hand, the total integral intensity of the NiO peaks increases with an increasing of nanoparticles concentration from spectrum to spectrum, and the total integral intensity of the Vulcan peaks decreases, but at NiO concentrations of 40, 50, and 80%, this tendency is violated.

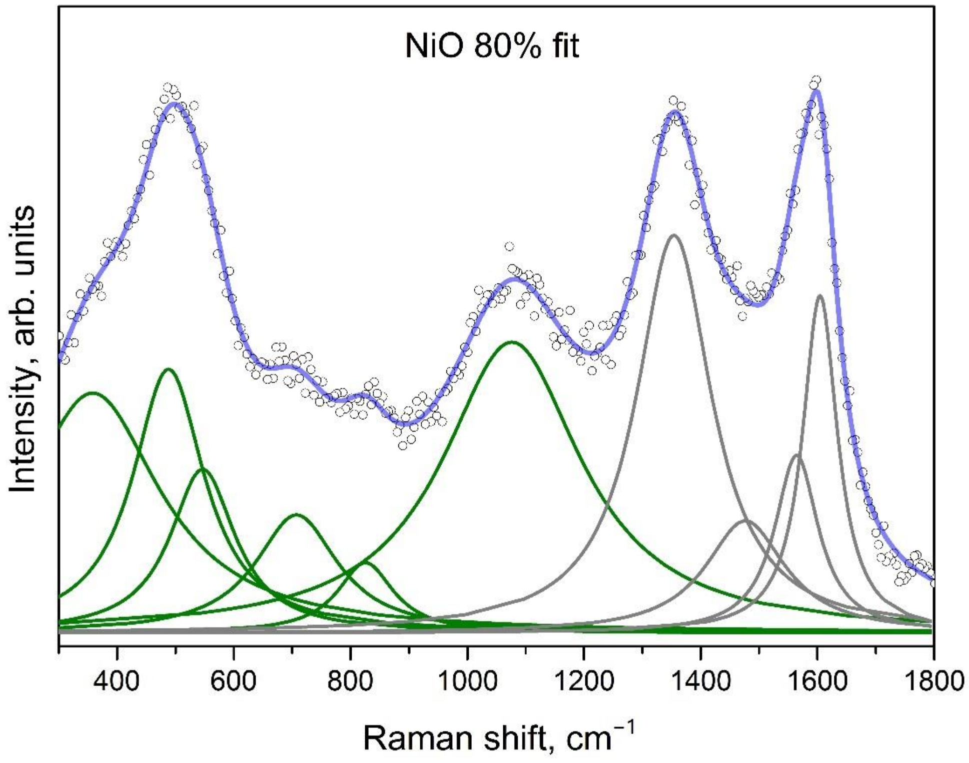


**Figure S5.** Decomposition into components of the NiO / Vulcan spectrum (NiO concentration—80%). Empty circles and blue curve correspond to experimental data, green curves—to NiO peaks, gray curves—to Vulcan peaks.
